# Diagnostic accuracy of *Fusobacterium nucleatum* IgA and IgG ELISA test in colorectal cancer

**DOI:** 10.1038/s41598-021-81171-1

**Published:** 2021-01-15

**Authors:** Melike Kurt, Zeki Yumuk

**Affiliations:** Department of Medical Microbiology, Kocaeli University Faculty of Medicine, 41380 Kocaeli, Turkey

**Keywords:** Cancer screening, Adaptive immunity, Bacteria

## Abstract

The colorectal cancer is a serious health problem. The diagnosis of the disease mostly relies on an invasive procedure. A non-invasive diagnostic test such as an immunoassay, may facilitate diagnosis of colorectal cancer. The purpose of the study was to evaluate the use of antibodies against *Fusobacterium nucleatum* in the diagnosis of colorectal cancer (CRC). Totally 78 patients in three groups were included in the study. *F. nucleatum* in the tissues was detected using quantitative polymerase chain reaction assay. *F. nucleatum* IgA and IgG were measured using enzyme linked immunosorbent assay. *F. nucleatum* was detected in 86.7% and 73.1% cases of CRC and precancerous-benign colon disease (P-BCD), respectively. The OD values from *F. nucleatum* IgA and IgG ELISA tests were higher in CRC group compared with healthy individuals. The sensitivity of IgA ELISA test varied between 31.8 and 95.5% depending on the chosen cut-off values. The positivity rate of antibodies in patients with high amount of *F. nucleatum* in tissue was significantly greater than in the negative group. The *F. nucleatum* IgA and IgG antibodies in CRC were higher than the ones in healthy controls but the discriminative ability of the ELISA test was not adequate to be considered as a diagnostic tool.

## Introduction

Colorectal cancer (CRC), an important threat to human health, is one of the most common cancer in the world^1^. As the cancer is a multifactorial disease, the pathogenesis of CRC is complex. Human gut hosts highly diverse and complex microbial community including virus, fungi and bacteria^2^. A disturbance in the microbiome structure such as loss of microbial diversity and beneficial microorganisms may lead to cancer development^3^. Some residents of microbiota in gut such as *Fusobacterium* spp., *Escherichia coli* and *Bacteroides fragilis* are suspected to play role in CRC^1^. Although there is a small amount of *F. nucleatum* found in the gut microbiota, it is supported that it may cause infection and consequently cancer in the colon tissue^4^.

The microbial community in the colon may influence the immune system development of host through their metabolites such as butyrate and retinoic acid. *F. nucleatum* promotes CRC through several virulence mechanisms such as invasion and modulation of host immune response^5^. The immune system can produce antibodies to keep host from invasion of microorganisms in colon. Those antibodies are sometimes extremely useful for the diagnosis of diseases especially when the pathogen organism cannot cultivate. Antibody tests are particularly important laboratory tools for the diagnosis of infectious disease because they are reliable and easy to use.

The purpose of the study was to evaluate the use of antibodies against *F. nucleatum* in the diagnosis of CRC. The reference tests were a colonoscopic examination and pathological evaluation of biopsy material.

## Results

### Patients and samples

A total of 78 patients were placed in three groups (22 CRC, 35 P-BCD and 21 HC). The patient characteristics are shown in Table 1. The average age was 59.7 ± 15.9 years in the study population and there were 39 females (50%). Median CRC tumor size was 4.5 cm (range, 2.0–9.5 cm) and tumor size exceeding 4.5 cm was observed in 7 patients (31.8%). CRC tumor was in the sigmoid colon in 8 patients (36.4%), at the hepatic flexure in 1 (4.5%), in the ascending colon in 2 (9.1%), in the cecum in 1 (4.5%), in the descending colon in 2 (9.1%) and in the rectum in 8 patients (36.4%). According to another definition, in 18 (81.8%) CRC cases, tumor was on distal colon (female: 8, 44.4%; mean age: 62.2 ± 14.2) and in 4 (18.2%) CRC cases, tumor was on proximal colon (female: 3, 75.0%; mean age: 82.0 ± 4.9).

**Table 1:** The study groups and patient characteristics.

Groups	Total	Female/male ratio (%)	Age (years ± SD)
All groups	78	50.0	59.7 ± 15.9
Colorectal cancer (CRC)	22	50.0	65.8 ± 15.1
Precancerous—Benign colon disease (P-BCD)	35	45.7	57.9 ± 14.8
Healthy controls (HC)	21	57.1	55.3 ± 17.9

### The amount of *F. nucleatum* DNA in tissue

**Figure 1:**
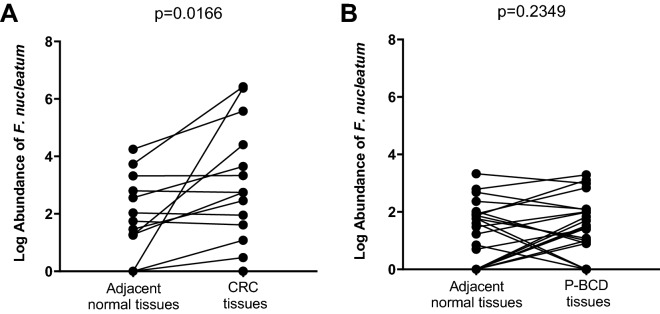
Log abundance of *F. nucleatum* DNA in tissues (15 CRC and 26 P-BCD patients). **(A)** CRC patients **(B)** P-BCD patients.

As shown in Table 2, 41 paired (15 CRC and 26 P-BCD) samples were analyzed using qPCR assays. The *F. nucelatum* DNA was detected in CRC and P-BCD. The detection rate of *F. nucleatum* was higher in CRC compared with P-BCD. In 10 (66.7%) CRC patients, both abnormal and adjacent normal tissues were positive for *F. nucleatum*. In 3 CRC cases (20%), *F. nucelatum* was detected only in abnormal tissues. In 2 CRC cases (13.3%) abnormal and adjacent normal tissues were both negative. In 3 P-BCD cases (11.5%), *F. nucleatum* was detected only in adjacent normal tissue. In CRC patients (Fig. 1a), the amount of *F. nucleatum* DNA in abnormal tissue was significantly greater (p = 0.0166) than in adjacent normal tissue. However, there was no significant difference (p = 0.2349) found in P-BCD patients (Fig. 1b) between abnormal and adjacent normal tissues.

**Table 2:** *F. nucleatum* detection rate in tissues using quantitative polymerase chain reaction.

Groups	Fusobacterium detection rate, %		
	Abnormal tissue	Adjacent Normal tissue	Abnormal and adjacent normal tissues both
All groups	78.0 (32/41)	61.0 (25/41)	53.7 (22/41)
Colorectal cancer	86.7 (13/15)	66.7 (10/15)	66.7 (10/15)
Precancerous—Benign colon disease	73.1 (19/26)	57.8 (15/26)	46.2 (12/26)

### Analysis of antibodies level against *F. nucleatum* by ELISA test

**Figure 2:**
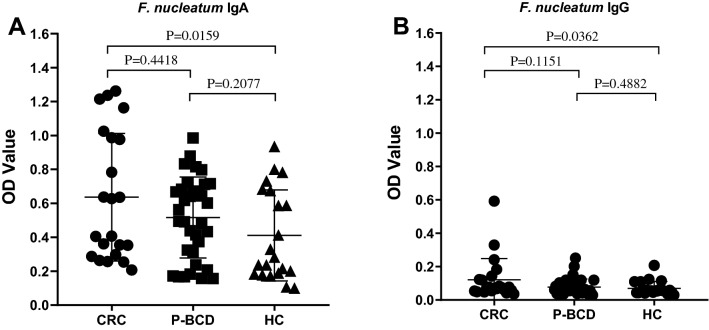
Comparison of OD values from *F. nucleatum* IgA and IgG ELISA in sera of CRC (n = 22), P-BRC (n = 35) and HC (n = 21) groups. Symbols indicated individual OD value; horizontal lines indicated mean values ± SD. **(A)** *F. nucleatum* IgA. **(B)** *F. nucleatum* IgG.

As shown in Fig. 2, 78 serum samples (22 CRC, 35 P-BCD and 21 HC) were analyzed using *F. nucleatum* IgA and IgG ELISA test. For each samples ELISA was run in triplicate. Average of optical density (OD) values were recorded. CV of the ELISA was determined below 20%.

The OD values from *F. nucleatum* IgA and IgG in CRC patients were significantly greater than in HC. There were no significant differences found in *F. nucleatum* IgA and IgG OD values between P-BCD and HC groups. The OD values were lower in the group of distal CRC patients in comparison with proximal CRC patients. With respect to IgG, the difference was statistically insignificant (proximal 0.175 ± 0.133 vs distal 0.109 ± 0.127, p = 0.3420). In case of IgA, the difference was more considerable but, still statistically insignificant (proximal 0.809 ± 0.497 vs distal 0.598 ± 0.350, p = 0.2622).

### Diagnostic accuracy of *F. nucleatum* IgA and IgG ELISA test in CRC

**Figure 3:**
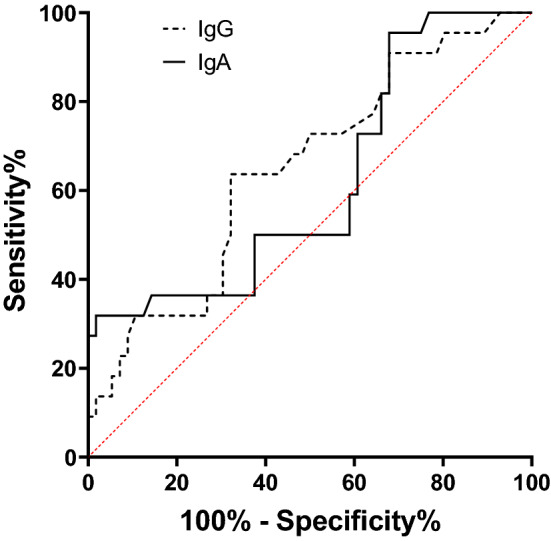
ROC curve for *F. nucleatum* IgA and IgG ELISA tests using serum from colorectal cancer patients and healthy controls. The reference tests were colonoscopic examination and pathological evaluation of biopsy material. IgA AUC = 0.6185, IgG AUC = 0.6481.

The global measure of diagnostic accuracy of *F. nucleatum* IgA and IgG ELISA tests were calculated using the AUC of ROC curve in Fig. 3. AUC of IgA and IgG ELISA test was 0.6185 and 0.6481, respectively. At the ROC curve and Youden index analysis it was found that the optimal cut-off value, the value providing the best tradeoff between sensitivity and specificity, for the identification of CRC was 0.246 and 0.047 for IgA and IgG, respectively (cut-off 1, first and third row of Table 3). However, the cut-off values corresponded to low specificity and low positive likelihood ratio. A relatively higher likelihood ratio was calculated when cut-off was 0.868 and 0.133 for IgA and IgG, respectively (cut-off 2, second and fourth row of Table 3).

**Table 3:** Diagnostic accuracy of *F. nucleatum* IgA and IgG ELISA test in colorectal cancer.

Test	Sensitivity (%)	Specificity (%)	Positive predictive value (%)	Negative predictive value (%)	Positive likelihood ratio	Approximate change in disease probability (%)	Youden’s index (*J*)
**IgA**							
Cut-off 1 (0.246)	95.5	47.6	65.6	90.1	1.8	13.5	0.43
Cut-off 2 (0.868)	31.8	95.2	87.5	57.1	6.6	38.5	0.27
**IgG**							
Cut-off 1 (0.047)	90.9	33.3	58.8	77.8	1.4	10.5	0.24
Cut-off 2 (0.133)	22.7	95.2	83.3	54.1	4.7	28.2	0.18

### Association between serum levels of IgA and IgG antibodies and the amount of *F. nucleatum* DNA within the tissue

To investigate the association between serum levels of IgA and IgG antibodies and the amount of *F. nucleatum* DNA within the tissue, cases (13 CRC and 26 P-BCD) with detectable *F. nucleatum* DNA were categorized as low (< 50 percentile) versus high (≥ 50 percentile) based on the median cut point amount of *F. nucleatum* DNA, while cases without detectable *F. nucleatum* were categorized as negative. The positivity rate of IgA ELISA (cut-off, 0.246) in the high percentile group was significantly (p = 0.0474) greater than in the negative group (Table 4). There were no significant differences found in IgA and IgG between low percentile and negative groups. Although high amount of *F. nucelatum* DNA were detected in P-BCD tissue of 3 patients, one was negative for IgA (cut-off: 0.246) and 2 were negative for IgG (cut-off, 0.047).

**Table 4:** Association between *F. nucleatum* in colon tissue and positivity rate of antibody tests in serum.

ELISA test	No of positive cases	No of cases with detectable *F. nucleatum* in tissue (%)		
		Negative (zero, n = 9)	Low (< 50 percentile, n = 15)	High (≥ 50 percentile, n = 15)
**IgA**				
Cut-off 1 (0.246)	31	5 (16.1)	12 (38.7)	14 (45.2)
Cut-off 2 (0.868)	7	2 (28.6)	2 (28.6)	3 (42.9)
**IgG**				
Cut-off 1 (0.047)	28	7 (25.0)	8 (28.6)	13 (46.4)
Cut-off 2 (0.133)	6	2 (33.3)	0	4 (66.7)

The positivity criterion is the cut-off that separates normal value (disease free) from abnormal values (indicative of disease).

## Discussion

CRC is a serious public health problem^1^ and diagnosis mostly relies on an invasive procedure such as colonoscopy exam. A reliable non-invasive test biomarker may facilitate diagnosis. In this study, the use of antibodies against *F. nucleatum* in the diagnosis of CRC was evaluated. According to the ROC curve and Youden’s index analysis diagnostic accuracy of test was poor. AUC is global measure of diagnostic accuracy and Youden’s index is valuable when identical load is given to sensitivity and specificity. From a clinical perspective, LR may have powerful properties and are linked to posttest probabilities. Depending on chosen cut-off value, the approximate change in probability of diseases could reach as high as 38.5% (cut-off: 0.868; LR + : 6.6) in CRC patients with IgA ELISA.

*Fusobacterium* has a role in CRC pathogenesis^6,7^. However, *F. nucleatum* antibodies for the diagnosis of CRC were rarely investigated^8,9^. In a multi-center study, no relationship was found between CRC and *F*. *nucleatum* antibodies with prediagnostic serum samples from 485 colorectal cancer cases and 485 matched controls^8^. Positive *F.nucleatum* antibody test results in healthy controls might be related to antibody responses resulted from other infection sites such as oral^10^. *F. nucleatum* is part of gut microbiota, the immune system can produce antibodies to keep host from invasion of *F. nucleatum* in colon. The amount of IgA produced in association with CRC was greater than IgG. The IgA were found more sensitive and specific than IgG. IgA plays an important role in the immune function of mucous membranes. In a previous study, AUC of *F. nucleatum* IgA and IgG ELISA tests in CRC were 0.704 and 0.645 for IgA and IgG, respectively and for an accurate diagnosis, carbohydrate antigen 19–9 and carcino-emryonic antigen tests were recommended in combination^9^.

In previous years, it has been identified that CRC tissues are generally infected *with F. nucleatum* but the rates between studies were highly divergent, ranging between 13% and 87.1%. In our study, the *F. nucleatum* detection rate in frozen CRC tissues was 86.7%. Similarly, Li et al. found *F. nucleatum* in 87.1% of frozen CRC tissues^11^. A report from USA demonstrated that the positive rate of *F. nucleatum* was 13% in formalin-fixed paraffin-embedded (FFPE) CRC tissue^12^. In Japan, 20 CRC cases were analyzed and the detection rate of *F. nucleatum* was 45% in FFPE CRC tissues^10^. One of the reasons for the different results among the current and the previous studies might be related to the tissue preparations used for the detection of *F. nucleatum*. The fixation process chemically alters the nucleic acid in a sample by inducing covalent DAN cross-linking and fragmentation. These alterations may reduce the efficacy of analysis using PCR and DNA sequencing methods. The varying rates of *F. nucleatum* in CRC tissues from different parts of the world might also be due to the characteristics of the study population demographic, environmental or genetic factors. Quantification of *F. nucleatum* in the stool of CRC patients was the concern of the most recent studies^13,14^. *F. nucleatum* was associated with the metastasis^11^ and the prognosis of CRC^12^. *F. nucleatum* is a new marker either when being quantified alone or combined with other bacteria. All those studies aimed to find either a non-invasive diagnosis of colorectal neoplasia or a successful therapy option. However, the etiology of cancer is multifactorial and the transformation of a human cell into a cancer cell is not straightforward.

There are a few limitations of this study. With the use of frozen tissue specimens and storage may influence the quantitative PCR assay to detect microorganisms. In addition *to F. nucleatum*, the other member of gut microbiota might be included to the study. In general, the study data reflects characteristics of geographic properties. Further studies with larger populations are compulsory to interpret the study results in detail. Although, performing sample size calculation during the planning phase of the study makes certain that the outcome might instructive, using too many samples may be considered unethical. To estimate the proper sample size, conducting a formal power analysis would be essential. In this study, ELISA was preferred to measure antibodies to *F. nucleatum* in the patient’s sera. ELISA has low cost and can easily be performed and automatized. The limited performance of the ELISA test might be related to the preferred antigen. Finally, we have no pathological data regarding the colon polyps to further study whether it has a relationship with IgA and IgG levels in P-BCD patients.

Although the discriminative ability of the *F. nucleatum* IgA ELISA test was poor, some measures largely depend on disease prevalence, and all of them are sensitive to the spectrum of the disease in the population studied. Referring to a specific cut-off, the probability of CRC for positive IgA ELISA increased considerably.

## Methods

### Patients and samples

Patients were selected randomly from the Gastroenterology and General Surgery of a university hospital between May 2018 and August 2019. All subjects were adult and informed consent was obtained from all subjects. All experiment protocols were approved by the Kocaeli Faculty of Medicine Clinical Research Ethical Committee, Turkey (29.03.2018-2018/134). All methods were carried out in accordance with Declaration of Helsinki. Patients underwent outpatient colonoscopy for colorectal cancer screening were enrolled and patients in whom colorectal cancer (CRC) or precancerous-benign colon disease (P-BCD) such as benign polyps, Crohn disease and ulcerative colitis were detected by a colonoscopy exam were included to the study. Patients who had ongoing cancer therapy or had a surgery for cancer treatment were excluded. Patients whose colonoscopy revealed no sign of CRC or P-BCD were assigned as healthy controls (HC).

Just before colonoscopy exam serum was acquired and at the time of colonoscopy exam tissue samples (biopsies) were obtained. Serum and tissue samples both were stored immediately at − 80 °C, until their analysis.

### Nucleic acid extraction and real-time PCR (RT-PCR)

According to the procedure described by Moen et al.^15^, the biopsies were extracted with DNA Mini Kit (Qiagen, Hilden, Germany). To measure concentration and purity of the extracted DNA, NanoDrop 1000 Spectrophotometer (Thermo Fisher Scientific, Waltham, MA, USA) was used. Microbial DNA qPCR Assay Kit (GeneGlobe Id: BBID00161A, Catalog No: 330033, Qiagen) was used according to the instructions of the manufacturers. The PCR reactions were performed with primers targeting the 16 s rRNA gene of *F. nucleatum* (Gene bank Acc. FJ471654.1). The reaction mixture was amplified on a Corbett Rotor-Gene Q apparatus (Qiagen) for 10 min at 95 ∘C and 2 min at 60 ∘C. Amplification, detection and data analysis were performed by using Rotor-Gene (Qiagen).

### Bacteria cultures

*Fusobacterium nucleatum* strain ATCC 25586 was purchased from Microbiologics, USA. *F. nucleatum* were grown 48 h in Columbia agar with 5% ship blood plate (BioMerieux, France), anaerobically at 37 °C.

### ELISA

Home-made manual ELISA tests were prepared and performed according to the procedure described elsewhere^9^. By an indirect whole cell ELISA, serum specific antibody to *F. nucleatum* IgA and IgG level was determined. Briefly, the 100 µl heated-inactivated *F. nucleatum* at a final concentration of 1 × 10^8^ CFU/ml in 0.05 M Na_2_CO_3_-NaHCO_3_ buffer was added to each well of ELISA plate and incubated overnight at 4 °C. To block each well, the plate was incubated at room temperature for 2 h with 200 µl of 1% non-fat dry milk in PBST. For the IgA ELISA test, serum samples were diluted 1:1000 and were incubated for 1 h at 37 °C. After washing 3 times, 100 µl ready to use anti-human IgA conjugate (Euroimmune, Germany) was added to the reaction well and was incubated at 30 min at 37 °C. For the IgG ELISA test, serum samples were diluted at 1:4000 in sample diluent and then diluted samples were incubated 1 h at 37 °C. After washing 3 times, 100 µl of ready to use anti-human IgG (Euroimmun, Germany) was added to the reaction well and was incubated for 30 min at 37 °C. In the final step, the substrate (tetramethylbenzidine) solution was added, and after 15 min of incubation the reaction was terminated using 2 M H_2_SO_4_. All reactions were read at an OD of 450 nm (reference wavelength 620–650 nm) by ELISA spectrophotometer (Triturus, Spain).

### Statistical analysis

The OD values and the abundance of bacteria between groups were compared by using Mann–Whitney U test. Positivity rate of ELISA test between *F.nucleatum* positive and negative in tissue groups were compared by using Fischer’s exact test. The discriminative ability of *F. nucleatum* IgA and IgG ELISA tests were quantified by several measures of diagnostic accuracy such as receiver operating characteristic (ROC) curve, area under the curve (AUC), Youden’s index, positive likelihood ratio, sensitivity, specificity, positive and negative predictive values^16–19^. The coefficient of variation (CV) was calculated with the following formula: CV% = (standard deviation/mean) × 100%.
